# Differential Antigen Expression Profile Predicts Immunoreactive Subset of Advanced Ovarian Cancers

**DOI:** 10.1371/journal.pone.0111586

**Published:** 2014-11-07

**Authors:** Kevin H. Eng, Takemasa Tsuji

**Affiliations:** 1 Department of Biostatistics and Bioinformatics, Roswell Park Cancer Institute, Buffalo, NY, United States of America; 2 Center for Immunotherapy, Roswell Park Cancer Institute, Buffalo, NY, United States of America; Philipps University, Germany

## Abstract

The presence and composition of lymphocytes characterizing an immune response has been connected to prognosis in advanced ovarian cancer. Our aim is to establish novel associations between prognosis and the expression of immune-related genes through a focused screen utilizing publicly available high-throughput assays. We consider transcriptome profiles from 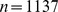 advanced ovarian cancer patients observed in four separate studies divided into discovery/validation sets (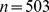/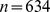). We focus on a subset of lymphocyte markers, antigen presentation and processing genes, T cell receptor associated co-stimulatory/repressor genes and cancer testis (CT) antigens. We modeled differential expression and co-expression using these subsets and tested for association with overall survival. Fifteen of 64 immune-related genes are associated with survival of which 5 are reproduced in the validation set. The expression of these genes defines an immunoreactive (IR) subgroup of patients with a favorable prognosis. Phenotypic characterization of the immune compartment signal includes upregulation of markers of CD8^+^ T-cell activation in these patients. Using multivariate model building, we find that the expression of 6 CT antigens can predict IR status in the discovery and validation sets. These analyses confirm that a genomic approach can reproducibly detect lymphocyte signals in tumor tissue suggesting a novel way to study the tumor microenvironment. Our search has identified new candidate prognostic markers associated with immune components and uncovered preliminary evidence of prognostic subgroups associated with different immune mechanisms.

## Introduction

Recent, high-throughput gene expression profiling studies in ovarian cancer have identified a theme of differentially regulated immune signaling molecules related to prognosis [1], [2]. The finding is consistent with mounting evidence that ovarian cancers are strongly immunogenic: spontaneous humoral and cellular immune reactions develop in response to disease [3], form characteristic epitopes [4], and are subject to complex up- and down-regulation by immune processes [5], [6]. The degree of tumor infiltration by host immune cells has been associated with good prognosis [7] as well as the balance of lymphocyte subtypes.

Subtypes of lymphocytes have diverse functions related to antigen recognition and immune suppression [8] and are thought to indicate a dynamic and evolving response to cancer [9]. For example, an increase in CD8^+^ T cells was found to be associated with good prognosis, but a rise in regulatory T cells was found to counteract this benefit [10]. Thus, studying components of the ovarian tumor microenvironment [11] is a critical angle for identifying prognostic associations [12].

However, few array-based expression studies have sampled both the host's reaction as well as the tumor. Most large observational studies [1], [2], [13], [14] considered only tumor tissue because their goal was prognostic modeling; immune associations were made *post hoc* based only on enrichment inferred from a small number of significant immune genes. Other array-based studies that focused on immune characterization were small and observational (n = 38) [15] or case control (n = 25, n = 25) [16]. These studies showed positive associations between lymphocyte-specific markers and prognosis implying that the sampled tissue contained some microenvironment signal.

Recent work in expression arrays is able to computationally separate tumor, stromal and immune components of these complex tissues. For example, the ESTIMATE algorithm [17] uses 141 genes to estimate the fraction of expression signal attributable to the immune compartment. While this approach is useful for eliminating the noise due to impure samples, this study noted that the immune signals did seem to carry mild association with clinical outcomes. Based on the presence and potential prognostic value of immune markers, we conjecture that the apparent associations can be traced to residual lymphocyte RNA and that further analysis of these markers can be attributed to signal from the microenvironment. As such, we might simply model markers that we suspect are highly likely to be immune-specific *a priori*.

We rely on two large cohorts of public data organized into a discovery set and a validation set: 503 biobanked high-grade serous ovarian cancer samples from the Cancer Genome Atlas (TCGA) [2] form a focused training and exploration set and a more clinically-representative mix of 634 samples from 3 Gene Expression Omnibus (GEO) studies [1], [13], [14] form the validation set. The clinical features of the data are described in their original papers, but briefly, all of the patients in these studies have advanced, primary ovarian cancer (a small number of primary peritoneal and fallopian tube in the validation set) and received adjuvant platinum and taxane treatments following surgery.

We study first the specificity of immune-related genes to lymphocyte tissues and then explore univariate prognostic associations. Using clustering algorithms, we identify a subset of cancer cases with high adaptive immunity signals and we show that this subset can be predicted by the tumor expression of cancer-testis antigens. Taken together, these results imply that significant prognostic value remains untapped in the tumor microenvironment.

## Methods

### Clinical and Gene Expression Data

TCGA is a biorepository study of 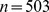 high-grade serous ovarian cancers from multiple centers in the United States and is described extensively in the original article [2]. Relevant to our analysis, this study strictly included ovarian primaries and papillary serous histologies. Samples were originally assessed as Stage III-IV and Grade 2,3 (later re-staged by a TCGA pathologist) and the patient received adjuvant platinum and taxane based chemotherapy. We adopt the view that these cases reflect a biased but homogenous clinical presentation more likely to yield a consistent biological mechanism.

Three clinical datasets were downloaded from the NCBI GEO database and are described below. Table 1 is a summary of clinically relevant differences between the studies.

**Table 1:** Descriptive statistics for data sets used in this study.

	Discovery	Validation		
Study	TCGA [2]	Australian [14]	Japanese [1]	US [13]
n	503	240	260	134
GEO Array Type	GPL570*	GPL570	GPL6480	GPL96
GEO identifier	NA	GSE9891	GSE32062	GSE3149
Age (Range)	59.7 (30–89)	60.2 (23–80)	NA	NA
Stage (% III, IV)	92%	5%	100%	NA
Grade (% 3,4)	87%	61%	50%	NA
Residual Disease (% None)	23%	27%	40%	NA
Neoadjuvant (% Yes)	0%	7%	0%	NA
Median Months OS	44 (40–48)	44 (38–57)	60 (50–80)	74 (35–98)
Median Months PFS	18 (15–19)	15 (14–18)	19 (18–23)	NA

^*^ TCGA uses 3 array types. Only the Affymetrix array was used for completeness.

GEO:GSE9891 is an Australian observational study [14] of 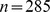 mostly serous (227) ovarian cancer including some peritoneal (34) and fallopian (5) primaries. Conditioning on patients receiving platinum/taxol and removing the LMP samples, we analyze 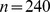 samples. For reproducibility purposes, we work with normalized data from GEO.

GEO:GSE32062 is a Japanese observational study [1] of 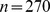 samples including 10 control samples, 193 recurrences, 121 deaths yielding 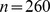 samples to analyze. An adjuvant platinum/taxol regimen was recorded for all patients.

GEO:GSE3149 is an observational study [13] of 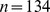 arrays after combining redundant ones. Significant work has been have been published on the difficulties in the original analysis of this data [18]; we have implemented the recommended similarity checks and averaged arrays when they appear to come from the same patient. This analysis begins with the GEO banked data and should be immune to data conversion problems.

A biological dataset, NCI-60 cell line data were taken from GEO:GSE5846 [19] where all of the cell lines were measured under untreated conditions. As positive controls, ovary cell lines, IGROV-1, OVCAR-3, OVCAR-4, OVCAR-5, OVCAR-8, SK-OV-3 and NCI/ADR-RES; should express no immune markers. For negative controls, we also study leukemia lines CCRF-CEM, HL-60(TB), MOLT-4, RPMI-8226, SR, and K-562.

### Candidate Immune-related and Cancer-Testis Antigen Genes

Candidate genes were selected from KEGG:Antigen processing and presentation (hsa04612) focusing on surface receptors and genes involved in plasma membrane transport. Surface markers and co-regulatory molecules of the T-cell receptor signaling pathway (hsa04660) were added, excluding the internal signaling mechanisms (for example: phospholipases, secondary messengers, and the kinase cascades) as these are non-specific and related to other signaling pathways. Cancer-testis antigen genes were defined based on overlap with the CT Database [20]. The full gene list is provided in Tables S1 and S4.

All genes were aligned using official gene names mapped using the provided GEO platform annotation (i.e., an associated GPL file) and R package hthgu133a.db-2.8.0 [21]. Expression values were scaled and centered for comparability across genes. Note that the highly variable parts of the T-cell receptor (TCA, TCB) cannot be measured on the hybridization-based oligonucleotide arrays.

### Computational Methods

Univariate associations were performed with proportional hazards regression. No time truncation, to reduce the effect of long survivors, was performed. Significance was cut at 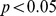 and was adjusted for multiple testing unless otherwise noted. The reported FDR calculation is based on the expected number of false positives assuming all tests are null.

Clustering analysis in the TCGA data is based on complete hierarchical clustering under Euclidean distance. Subgroups were picked by splitting the tree at 4 leaves based on visual inspection and a within/between group sum of squares criterion. Cluster centers were used to seed a k-means clustering algorithm in the validation data and the p-value is reported for a study-stratified 4-group log-rank test.

The volcano plot uses literature-based surface markers and co-regulators based on a simple t-test. Highlighted genes were significant above the Bonferroni p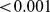 mark and had biologically extreme fold change values.

Partial correlations were computed using the class averages for genes assigned to CD4, CD8, CD3, MHCI and MHCII-based sets. The GeneNet algorithm [22] using FDR 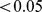 and hard thresholding at absolute correlation 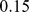 was used to infer Gaussian graphical models.

To infer the missing nodes in the GGM, a hidden node must satisfy three properties. Using MHCII - X - CD8 as an example: MHCII and CD8 must be conditionally independent given Gene X (p
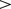
0.05); MHCII and Gene X must be conditionally dependent given all other genes (Bonferroni p
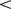
0.05); CD8 and Gene X must be conditionally dependent given other genes (Bonferroni p
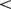
0.05). Each of these can be reduced to a p-value statement using standard linear model theory and added variable analysis.

Predictions from a multivariate logistic regression model were categorized into immunoreactive class calls based on thresholding the predicted value from the model. This threshold was chosen using the sensitivity/specificity intersection point across all validation datasets.

All statistical analysis was performed in the R statistical programming language.

## Results

### Expression of genes specific to lymphocytes is measurable in tumor samples

**Figure 1:**
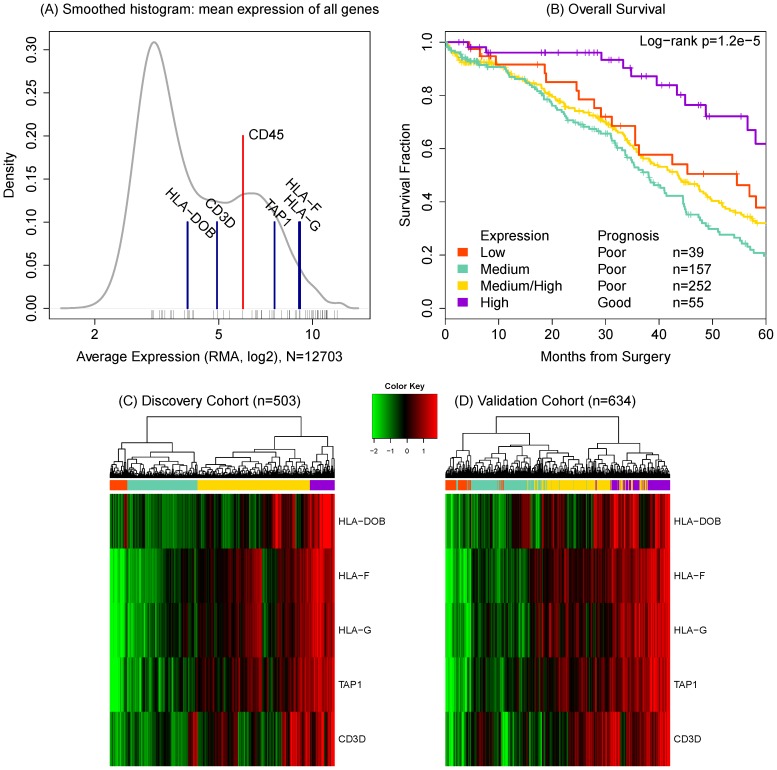
Mean relative expression of the selected genes confirms that they are all expressed in the samples (A). Black ticks indicate the mean expression of immune-related genes. CD45 is highlighted as a lymphocyte specific marker indicating the presence of lymphocytes. Subgroup-based survival estimates (B) are shown for the TCGA data based on hierarchical clustering of relative expression of selected T-cell genes (C and D). The four color bar on the left identifies the subgroups. The purple subset (n = 55, 11%) represents a significant survival benefit associated with the expression of all five genes.

We observed that selected lymphocyte specific markers are present in measurable quantities in sampled tumor tissue from our discovery set. The distribution of average expression (Figure 1A) shows the typical multimodal pattern in expression arrays: the lower peak reflects the background noise for genes that are not expressed in the sample and the higher peak represents signal. The lymphocyte marker CD45 is in the signal peak and is relatively highly expressed (Table S2). We conjecture that a measurable portion of the cells in each sample contain lymphocyte RNA which would represent the tumor microenvironment.

Comparison with the GeneAtlas tissues [23] confirms that the nominal action of these expression array probes is lymphocyte-specific. We considered probe expression in ovarian NCI-60 cell lines [19] to exclude the possibility that genes are normally expressed in tumor tissue (Table S2). Considering the mean quantile of expression versus other probes, surface markers CD64, CD1D, CD14, CD33, CD8A, CD16b, CD45 maintain a low level in cell lines versus microdissected tumor (all lower than the 35th quantile, p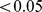). The near absence of IL6, IL12, IFNB1, and IFNG (between the 2nd and 25th quantile) suggests that the ability to measure cytokine signaling is lost in this in vitro system; notably, the IL12 receptor is measurable, so surface markers do appear. The exception to this pattern is that CD4 is over-expressed (51st and 61st quantiles in tumor and cell line samples respectively).

This establishes that a reproducible immune signal can be measured in sampled tumor tissue and we proceed assuming that these markers form a representative cross-section of tumor and immune system interaction.

### Reproducible associations between candidate genes and overall survival

Of the 64 candidate genes, 15 have univariate associations (score test p-value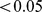) with overall survival (OS) following surgery and primary chemotherapy in the TCGA study (Table S1). A further 5 of these can be validated in the withheld independent studies (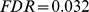).

The validated set can be organized by function: major histocompatibility complex (MHC) I genes, HLA-F, and HLA-G; an MHCII gene, HLA-DOB; the MHCI associated transporter, TAP1; and the co-receptor complex subunit, CD3D (TCR-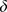). Increasing expression of each of these transcripts is associated with better survival (Table 2). T-cell related genes are highly correlated, which likely accounts for an indirect but positive association between the suppressor HLA-DOB and survival (Univariate HR = 0.74, 95% CI: 0.65–0.85, p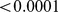). We will examine the multivariate expression of these genes next.

**Table 2:** Validated immune genes related to overall survival and their correlation structure.

			Correlation			
Gene Name	HR (95% CI)	p-value	HLA-G	HLA-DOB	TAP1	CD3D
HLA-F	0.89 (0.79–1.00)	0.0429	0.95	0.51	0.84	0.60
HLA-G	0.88 (0.79–0.99)	0.0282		0.48	0.81	0.54
HLA-DOB	0.74 (0.65–0.85)	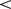 0.0001			0.57	0.41
TAP1	0.88 (0.79–0.98)	0.0238				0.58
CD3D	0.85 (0.75–0.96)	0.0087				

Using a hierarchical clustering algorithm, the discovery set patients can be divided into four groups (Figure 1B) associated with OS (p = 1.2e-05). Represented in the heatmap in Figure 1C, the simultaneous expression of all five genes (colored purple) confers the most benefit. The simultaneous expression of all five genes is consistent with T cell activation, so we deem the high expressing subgroup an immunoreactive (IR) subset.

The degree of expression is not associated with variation in poor prognosis: the low expression of all genes (orange) does no worse than a heterogenous pattern of high and medium expression (yellow) or uniformly medium expression (green subgroup). This suggests that the deficient expression of any one gene is sufficient to lead to poor prognosis.

We assign validation patients to their most similar discovery set subgroup by k-means clustering. In the second heatmap (Figure 1D), the patients (columns) are ordered by hierarchical clustering within the validation data set. So, the clustered subgroups are strengthened by the observation that the class labels are preserved in the validation set.

In the discovery data, the survival benefit for the high expressers (n = 55, 10%) is a median of 70.9 months (95% CI: 58.1–98.0) versus 41.4 (36.9–45, p = 3.5e-05) OS; the median progression free survival (PFS) benefit is significant (p = 3.1e-04) at 30.4 (18.2–91.3) months versus 16.4 (14.7–18). This subgroup accounts for 5% of the deaths observed in the dataset and 7% of the recurrent cases. This benefit is weakly reproduced in the validation data: (p = 0.0335) with a difference of 64.0 months versus 51 months median OS. The PFS difference was not significant in the validation set.

### Association with immunoreactive subgroup and immune markers

The TCGA analysis confirmed a set of genetic subsets [2] identified in a previous study [14]. Our high expression subgroup (purple, n = 55) is associated with their IR subgroup (38/55, 69%) (p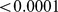) (Table 3). The orange subgroup (n = 39) is mostly proliferative (31/37, 84%). Both of these associations hold in the validation data with TCGA subtypes. Altogether, this suggests that the hierarchical clustering derived subgroup is meaningful and that it nuances the TCGA subtypes as the latter were found to have no significant survival associations. Note that our study focuses on the IR subset and we make no attempt to model the other TCGA subtypes.

**Table 3:** Association between derived subgroups and previously identified TCGA subgroups in discovery and validation sets.

Class	Purple	Yellow	Aqua	Orange
n	55	252	157	39
Overall Survival				p = 1.2e-05
median months	71	43	38	55
Progression-Free Survival				p = 6.7e-04
median months	30	18	15	17
Age				p = 6.2e-03
Mean Years	58	57	61	63
Stage				p = 2.8e-05
I/II	7	13	3	1
III/IV	48	239	154	38
Debulking Status				p = 3.2e-03
Optimal	17	51	21	13
Suboptimal	29	170	124	25
Platinum Status				p = n/s
Resistant	7	42	34	7
Sensitive	20	103	59	14
TCGA Class				p 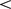 2.2e-16
Immunoreactive	38	59	7	0
Other	12	184	141	37
TCGA Class (Validation set)				p = 6.4e-11
Immunoreactive	35	27	8	0
Other	15	52	58	29

The colors are consistent with Figure 1. Note that only one study in the validation set had predicted TCGA classes and totals may not sum to 503 due to missing data.

**Figure 2:**
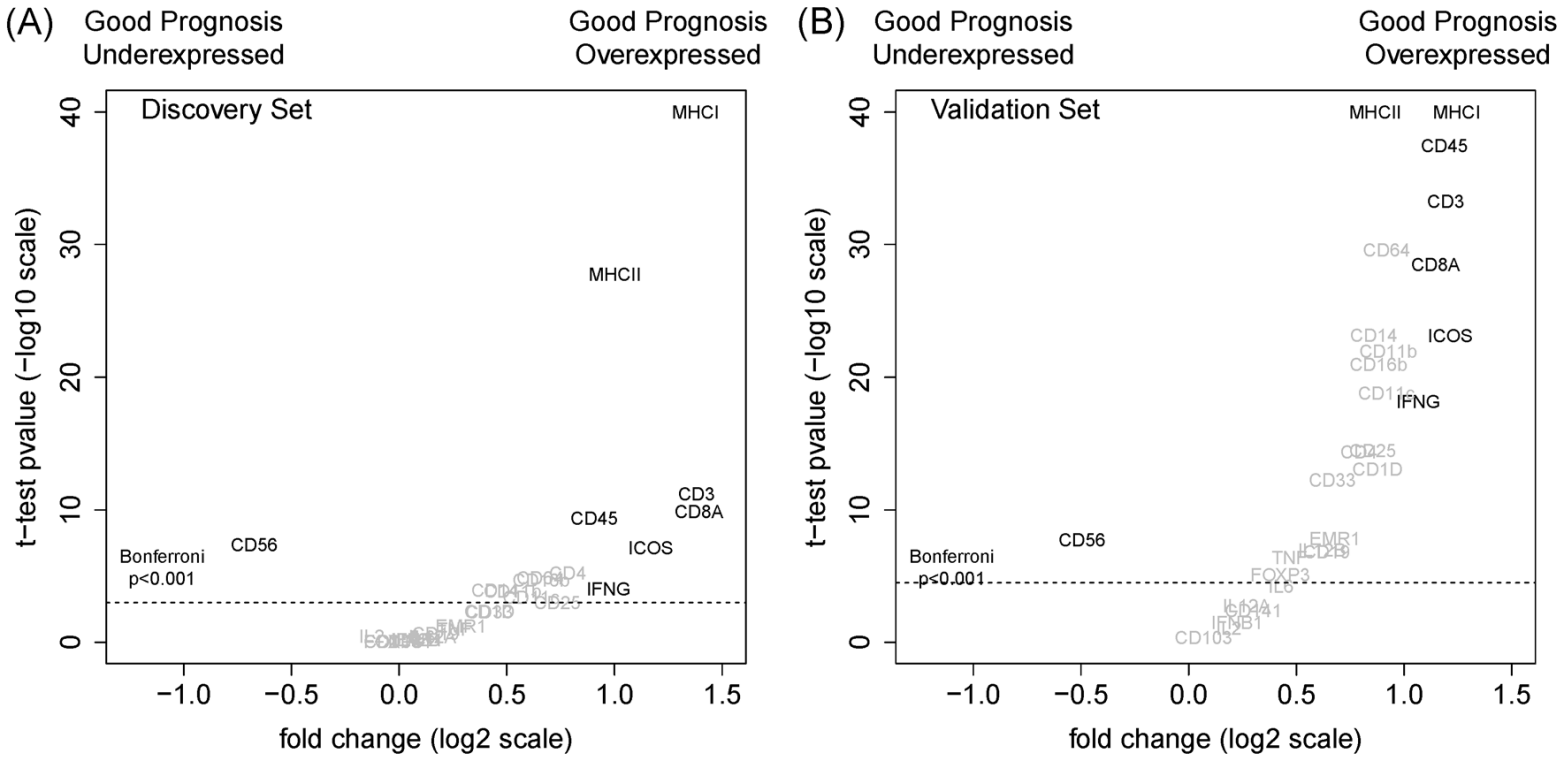
Differential expression of immunological markers in discovery (n = 503) and validation (n = 634) sets. Markers with strong biological and statistical significance (t-test Bonferroni p
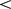
0.001) are chosen in the discovery set and highlighted in both plots.

We examined the differential expression of standard immunology markers in the good prognosis subgroup. Figure 2 plots the change in expression across prognosis subgroups versus the statistical evidence in both datasets. These markers imply an inflammatory response mediated by the recruitment of CD45^+^ cells: CD8^+^ cells and antigen-presenting cells (APC). No change in CD4 expression suggests that there is likely to be an latent background presence of CD4^+^ T-cells and that the different action is attributable to activation (represented by differential *ICOS* expression). Absent are the NK cells (CD56^+^) reinforcing the idea that the process is part of adaptive immunity.

We conjecture that these observations are consistent with an immunoediting concept. The good prognosis group represents equilibrium where a prevalence of APCs and activated CD4^+^ cells recruit CD8^+^ T-cells to maintain adaptive immunity.

### Prognostic co-expression suggests T-cell activation

**Figure 3:**
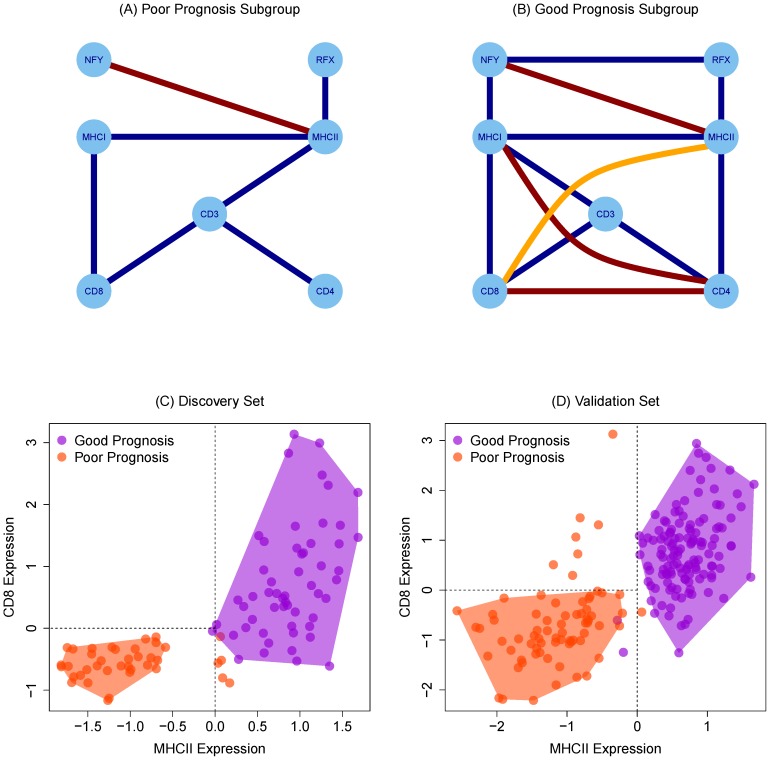
Partial correlation graphs indicating conditional relationships between genes in poor prognosis (n = 39)(A) and good prognosis (n = 55)(B) subgroups. Blue edges are positive associations and red are inhibitory associations. The highlighted orange edge can be removed by considering an independent set of genes. CD8 and MHCII array expression separate good and poor prognosis subgroups in discovery (C) and validation (D) data sets. MHCII expression is positively prognostic but appears in CD8 expressing samples. Shading is added for emphasis.

To infer functional interactions between immune-related genes, we performed a co-expression analysis which summarizes correlation in the IR subgroup and the poor prognosis subgroups graphically. Edges in these Gaussian graphical models (GGMs) represent statistical dependence between immune components (Figure 3A, 3B); positive dependence implies that both components are present and are likely to be interacting while negative edges imply mutually exclusive function or repression.

As in the differential expression analysis, CD4 and MHCII are correlated in the IR subgroup and not in the poor prognosis subgroups. Consistent with its reported function, RFX family (*RFX5*, *RFXANK*, *RFXAP*) of transcription factors' expression increases concordantly with the rise in MHCII expression [24]. In the IR subgroup, two edges imply anti-correlation between CD4 and MHCI which implies mutually exclusive function; and correlation between CD8 and MHCII (Figure 3C, 3D) which appears contrary to the specificity of MHC classes.

To explain this effect, we searched for missing genes whose inclusion in the graph would remove these edges. A missing link between CD8 and MHCII is *GZMA* a protease reflecting the activity of cytolytic T-cells. Between CD4 and MHCI we infer that CCR5, a cytokine receptor related to the activation of the T-cells, is missing. The common observation is that transcript associations likely need to be considered alongside measures of T-cell activation. The relevance of these genes is supported by the statistical significance of the transcriptome-wide search (adjusted 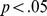) and the specificity of the selected genes' expression in lymphocyte tissues.

Focusing on CD8 and MHCII expression, we note that the good prognosis group can be defined by high MHCII expression where CD8 expression varies; CD8 expression is restricted when MHCII expression is low. Therefore, we hypothesize that MHC class II-mediated activation of CD4^+^ T-cells are required for infiltration by CD8^+^ T-cells that provide protection.

### Immunoreactivity predicted by cancer-testis antigen profiles

We investigated the association of the IR subgroup with the cancer testis (CT) family of antigens [20]. These genes are frequently over-expressed in cancer cells and induce spontaneous immune responses, which make them a primary target for immunotherapy [25]. In particular, CT antigen expression is believed to influence the IR subset by regulating T-cell responses in the ovarian tumor microenvironment. The discovery set arrays measured 98 CT antigens (Table S4) of which 3 were associated with the IR subset (Bonferroni p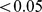): *CEP290*, *CTNNA2*, *TMEFF1*.

Because membership in the IR class is binary, we used logistic regression to model the multivariate set of antigens associated with IR status. Table S3 is the regression table for a model fit using BIC-based stepwise selection. Here, *CEP290*, *CTNNA2*, *TMEFF1*, and *TEX15* expression decreases the likelihood that a patient is in the IR class. Antigens *ZNF164* and *MAGEA3* increase the chance. Other than *CTNNA2*, which is twice as important as *MAGEA3*, the other genes have about the same effect. In the independent data, predictions from this model are strongly associated with the k-means derived associations (HR = 3.96, 95% CI: 2.59–6.14, p = 1.47e-11) tuned for equal sensitivity and specificity (0.66) given a moderate prevalence (140/634, 20.5%).

**Figure 4:**
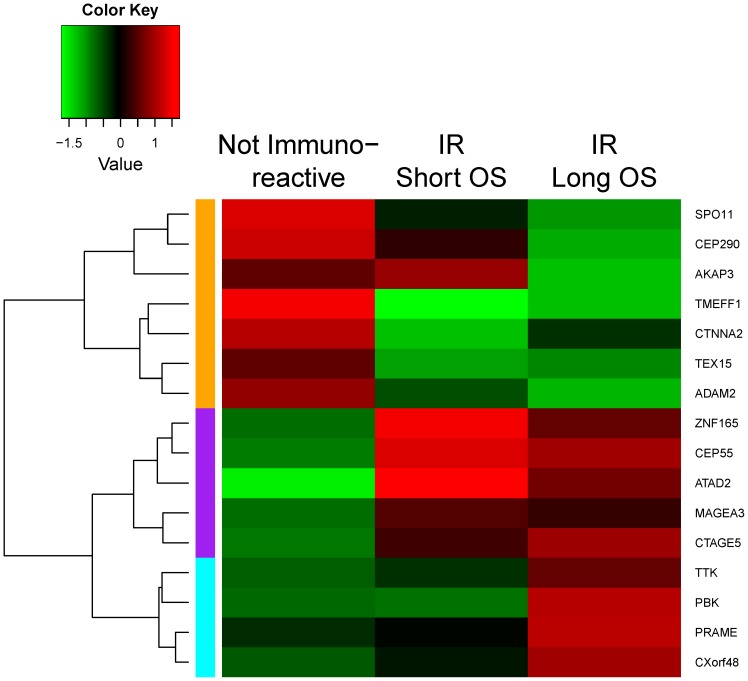
Cancer testis antigens associated with prognosis or immunoreactive (IR) subgroup can be divided into three classes: non-IR (orange), IR (purple) and immune stimulatory (cyan).

We further stratified patients in IR subgroup into good and poor prognosis based on OS to 33.5 months (overall study population median OS). A set of 16 antigens includes the union of the 3 IR subset genes and antigens associated with the difference in survival. Figure 4 is a heatmap comprising the mean expression of the 16 genes organized into three classes. The first class (orange) is expressed in non-IR cases and may reflect the activity of immunosuppressive elements. The second set (purple) is expressed in IR cancers but is unrelated to prognosis. The third (cyan) is expressed strongly in IR cases with good prognosis and may indicated immune stimulatory effects. A viable hypothesis might be to see if T cells responding to the second set of antigens (*ZNF165*, *CEP55*, *ATAD2*, *MAGEZ3*, *CTAGE5*) have regulatory phenotypes.

## Discussion

We have analyzed the expression of 64 T-cell co-receptor and antigen presentation/processing genes in advanced serous ovarian cancer using a standard univariate screen and an analysis of their co-expression. These analyses define a subset of ovarian cancer cases with prognostically meaningful expression associated with T-cell activation and a previously defined immunoreactive subgroup [2]. In contrast to previous work [17], [26], the subgroup can be identified reproducibly with just 5 genes versus over a hundred. This efficiency comes from our initial immunologic perspective. We reduce significantly the extraneous genes, but trade the ability to make a pan-ovarian cancer genetic characterization. As a result, we make no claim about or attempt to model non-immunologic signals.

We now have a small set of markers and antigens that may make translational and biomarker work more feasible for immunotherapy. With respect to the use of CT antigens, we have found a set which predicts non-immunogenic cancers (putatively, ones with low T-cell activation) and a set that might be targeted for blockade type immunotherapy. Both biological studies to verify regulatory activity of T cells in these cases and retrospective clinical studies may be the next investigative step. We speculate that the set of IR specific CT antigens stimulate immune responses (e.g., recruiting activated T cells), but are insufficiently immunogenic to induce tumor eradicating immune reaction. Alternatively, this group may prompt mixed effects: inducing immune responses while promoting tumor progression.

Inferentially, we have adopted a discovery/validation framework that allows us to make preliminary confirmations and to avoid over-interpreting high-dimensional artifacts. The data are limited by the nature of expression data from tumor samples; we rely on the conjecture that even after microdissection, assayed samples retain a portion of lymphocyte genetic material. Because this effect appears independently in multiple studies, these observations may be applicable to assays using this platform and are less likely to be subject to batch effect bias. Additionally, this work motivates the investigation of a structured way to analyze residual stromal signal from the tumor samples and therefore infer genetic interaction in the tumor microenvironment.

## Supporting Information

Table S1 **Univariate associations between genes and overall survival measured by score test p-value.**
(PDF)

Table S2 **Tissue specific quantiles of expression of lymphocyte specific markers.** Low quantiles imply relatively low or no expression.
(PDF)

Table S3 **Regression table for multivariate logistic regression model using antigen expression to predict immunoreactive class status.**
(PDF)

Table S4 **List of identified cancer testis (CT) antigens.**
(PDF)
