# Supplementary material for: Differential Antigen Expression Profile Predicts Immunoreactive Subset of Advanced Ovarian Cancers
Source: PLoS One. 2014 Nov 7;9(11):e111586. doi: 10.1371/journal.pone.0111586 (PMC4224408; doi:10.1371/journal.pone.0111586)
Supplement: Table S1 — Univariate associations between genes and overall survival measured by score test p-value. (PDF) [file pone.0111586.s001.pdf]

Table S1. Univariate associations between genes and overall survival measured by score test p-value.

Gene Symbol	Entrez ID	Gene Description	Discovery p-value	Validation p-value
B2M	567	beta-2-microglobulin	7.18e-01	6.36e-02
CALR	811	calreticulin	1.68e-02	2.69e-01
CANX	821	calnexin	6.88e-01	6.79e-01
CD4	920	CD4 molecule	1.55e-01	7.28e-02
CD74	972	CD74 molecule, major histocompatibility complex, class II invariant chain	7.82e-01	2.01e-03
CD8A	925	CD8a molecule	2.28e-01	2.25e-01
CD8B	926	CD8b molecule	4.37e-01	2.45e-02
CIITA	4261	class II, major histocompatibility complex, transactivator	3.56e-01	1.88e-03
CREB1	1385	cAMP responsive element binding protein 1	5.17e-01	2.44e-01
CTSB	1508	cathepsin B	8.31e-01	4.11e-02
CTSS	1520	cathepsin S	3.91e-01	8.78e-04
HLA-A	3105	major histocompatibility complex, class I, A	1.71e-01	1.54e-02
HLA-B	3106	major histocompatibility complex, class I, B	3.84e-01	3.43e-02
HLA-C	3107	major histocompatibility complex, class I, C	1.40e-01	1.77e-02
HLA-DMA	3108	major histocompatibility complex, class II, DM alpha	2.38e-01	9.21e-04
HLA-DMB	3109	major histocompatibility complex, class II, DM beta	5.54e-02	1.73e-02
HLA-DOA	3111	major histocompatibility complex, class II, DO alpha	2.16e-02	8.27e-01
HLA-DOB	3112	major histocompatibility complex, class II, DO beta	2.09e-05	2.17e-02
HLA-DPA1	3113	major histocompatibility complex, class II, DP alpha 1	7.09e-01	3.11e-03
HLA-DPB1	3115	major histocompatibility complex, class II, DP beta 1	4.50e-01	1.67e-02
HLA-DQA1	3117	major histocompatibility complex, class II, DQ alpha 1	2.32e-01	1.45e-01
HLA-DQB1	3119	major histocompatibility complex, class II, DQ beta 1	4.50e-01	4.23e-03

Table S1. Univariate associations between genes and overall survival measured by score test p-value, continued.

Gene Symbol	Entrez ID	Gene Description	Discovery p-value	Validation p-value
HLA-DRA	3122	major histocompatibility complex, class II, DR alpha	2.98e-01	4.77e-04
HLA-DRB4	3126	major histocompatibility complex, class II, DR beta 4	3.68e-01	6.07e-03
HLA-E	3133	major histocompatibility complex, class I, E	6.76e-02	2.10e-03
HLA-F	3134	major histocompatibility complex, class I, F	4.28e-02	5.21e-03
HLA-G	3135	major histocompatibility complex, class I, G	2.81e-02	1.93e-02
HSP90AA1	3320	heat shock protein 90kDa alpha (cytosolic), class A member 1	2.19e-01	3.16e-01
HSP90AB1	3326	heat shock protein 90kDa alpha (cytosolic), class B member 1	3.71e-02	5.24e-01
HSPA1L	3305	heat shock 70kDa protein 1-like	3.93e-03	2.85e-01
HSPA2	3306	heat shock 70kDa protein 2	4.00e-02	3.00e-01
HSPA4	3308	heat shock 70kDa protein 4	4.99e-01	5.22e-01
HSPA5	3309	heat shock 70kDa protein 5 (glucose-regulated protein, 78kDa)	1.08e-01	1.01e-01
HSPA6	3310	heat shock 70kDa protein 6 (HSP70B')	8.49e-01	9.41e-01
IFI30	10437	interferon, gamma-inducible protein 30	3.09e-01	3.85e-02
IFNG	3458	interferon, gamma	1.30e-01	7.77e-02
KIR2DL2	3803	killer cell immunoglobulin-like receptor, two domains, long cytoplasmic tail, 2	7.75e-01	9.03e-02
KIR2DL3	3804	killer cell immunoglobulin-like receptor, two domains, long cytoplasmic tail, 3	7.61e-01	4.33e-01
PDIA3	2923	protein disulfide isomerase family A, member 3	7.30e-01	3.49e-01
PSME1	5720	proteasome (prosome, macropain) activator subunit 1 (PA28 alpha)	4.09e-01	4.52e-05

Table S1. Univariate associations between genes and overall survival measured by score test p-value, continued.

Gene Symbol	Entrez ID	Gene Description	Discovery p-value	Validation p-value
PSME2	5721	proteasome (prosome, macropain) activator subunit 2 (PA28 beta)	1.43e-01	3.21e-06
PSME3	10197	proteasome (prosome, macropain) activator subunit 3 (PA28 gamma; Ki)	1.25e-01	5.69e-01
RFX5	5993	regulatory factor X, 5 (influences HLA class II expression)	9.32e-01	8.72e-01
RFXANK	8625	regulatory factor X-associated ankyrin-containing protein	6.27e-01	5.13e-01
RFXAP	5994	regulatory factor X-associated protein	5.32e-01	4.42e-02
TAP1	6890	transporter 1, ATP-binding cassette, sub-family B (MDR/TAP)	2.38e-02	9.79e-04
TAP2	6891	transporter 2, ATP-binding cassette, sub-family B (MDR/TAP)	2.46e-02	1.80e-01
TAPBP	6892	TAP binding protein (tapasin)	1.27e-01	6.83e-02
TNF	7124	tumor necrosis factor	1.26e-01	9.63e-01
CD3E	916	CD3e molecule, epsilon (CD3-TCR complex)	1.32e-02	4.16e-01
CD3G	917	CD3g molecule, gamma (CD3-TCR complex)	5.63e-04	2.92e-01
CD3D	915	CD3d molecule, delta (CD3-TCR complex)	8.71e-03	1.21e-02
CD247	919	CD247 molecule	2.96e-02	1.14e-01
PDCD1	5133	programmed cell death 1	1.64e-01	1.33e-01
CTLA4	1493	cytotoxic T-lymphocyte-associated protein 4	9.47e-02	2.40e-03
PTPRC	5788	protein tyrosine phosphatase, receptor type, C	3.01e-01	7.66e-02
CD28	940	CD28 molecule	9.70e-01	1.66e-01
ICOS	29851	inducible T-cell co-stimulator	1.62e-02	9.49e-02
CD40LG	959	CD40 ligand	5.51e-02	1.83e-01
