# Supplementary material for: Differential Antigen Expression Profile Predicts Immunoreactive Subset of Advanced Ovarian Cancers
Source: PLoS One. 2014 Nov 7;9(11):e111586. doi: 10.1371/journal.pone.0111586 (PMC4224408; doi:10.1371/journal.pone.0111586)
Supplement: Table S2 — Tissue specific quantiles of expression of lymphocyte specific markers. Low quantiles imply relatively low or no expression. (PDF) [file pone.0111586.s002.pdf]

**Table S2.** Tissue specific quantiles of expression of lymphocyte specific markers. Low quantiles imply relatively low or no expression.

| | Quantiles | | | |
|---|---|---|---|---|
| Gene | Tumor | Cell Line | p | Comments |
| IL12B | 0.50 | 0.021 | 0.0e+00 | |
| FCGR1A | 0.50 | 0.051 | 6.8e-232 | (CD64) |
| CD1D | 0.49 | 0.055 | 3.0e-56 | |
| IL12A | 0.48 | 0.093 | 1.2e-255 | |
| CLEC4A | 0.49 | 0.148 | 1.9e-210 | (CD303) |
| IFNB1 | 0.49 | 0.152 | 2.8e-199 | |
| FCGR3B | 0.51 | 0.153 | 1.7e-214 | (CD16b) |
| CD14 | 0.53 | 0.204 | 6.8e-104 | |
| IFNG | 0.48 | 0.205 | 2.8e-155 | |
| IL6 | 0.47 | 0.231 | 6.5e-16 | |
| EMR1 | 0.49 | 0.312 | 1.1e-21 | |
| ITGAM | 0.51 | 0.317 | 3.6e-71 | (CD11b) |
| CD19 | 0.48 | 0.318 | 8.1e-57 | |
| FOXP3 | 0.50 | 0.329 | 3.0e-46 | |
| PTPRC | 0.52 | 0.350 | 1.8e-11 | (CD45) |
| ITGAX | 0.50 | 0.377 | 2.8e-25 | |
| TNF | 0.48 | 0.390 | 5.4e-11 | |
| CD33 | 0.50 | 0.426 | 9.6e-07 | |
| CD8A | 0.50 | 0.472 | 3.2e-02 | |
| NCAM1 | 0.48 | 0.476 | 8.7e-01 | (CD56) n/s |
| THBD | 0.48 | 0.488 | 7.8e-01 | (CD141) n/s |
| IL2RA | 0.50 | 0.499 | 9.5e-01 | n/s |
| CD4 | 0.51 | 0.607 | 5.0e-16 | overexpressed |
| ITGAE | 0.50 | 0.918 | 5.7e-173 | overexpressed |
