# Supplementary material for: Differential Antigen Expression Profile Predicts Immunoreactive Subset of Advanced Ovarian Cancers
Source: PLoS One. 2014 Nov 7;9(11):e111586. doi: 10.1371/journal.pone.0111586 (PMC4224408; doi:10.1371/journal.pone.0111586)
Supplement: Table S3 — Regression table for multivariate logistic regression model using antigen expression to predict immunoreactive class status. (PDF) [file pone.0111586.s003.pdf]

Table S3. Regression table for multivariate logistic regression model using antigen expression to predict immunoreactive class status.

	Estimate	Std. Error	z value	$Pr(> \|z\|)$
(Intercept)	-2.8086	0.2462	-11.409	< 2e-16 ***
CTNNA2	-0.7965	0.3051	-2.610	0.009044 **
ZNF165	0.6294	0.1646	3.825	0.000131 ***
TMEFF1	-0.6014	0.1899	-3.167	0.001543 **
CEP290	-0.5942	0.1850	-3.212	0.001318 **
TEX15	-0.4828	0.1782	-2.710	0.006730 **
MAGEA3	0.3669	0.1192	3.079	0.002075 **

Significance codes: 0 *** 0.001 ** 0.01 . 1
