# Supplementary material for: Differential Antigen Expression Profile Predicts Immunoreactive Subset of Advanced Ovarian Cancers
Source: PLoS One. 2014 Nov 7;9(11):e111586. doi: 10.1371/journal.pone.0111586 (PMC4224408; doi:10.1371/journal.pone.0111586)
Supplement: Table S4 — List of identified cancer testis (CT) antigens. (PDF) [file pone.0111586.s004.pdf]

Table S4. List of identified cancer testis (CT) antigens.

Antigen	Location	CT.identifier	Class	Entrez ID	Gene Description
SPO11	20q13.2-q13.3	CT35	Not IR	23626	SPO11 meiotic protein covalently bound to DSB homolog
CEP290	12q21.32	CT87	Not IR	80184	Centrosomal protein 290kDa
AKAP3	12p13.3	CT82	Not IR	10566	A kinase (PRKA) anchor protein 3
TMEFF1	9q31	CT 120.1	Not IR	8577	transmembrane protein with EGF-like and two follistatin-like domains 1
CTNNA2	2p12-p11.1	CT 114	Not IR	1496	catenin (cadherin-associated protein), alpha 2
TEX15	8p12	CT42	Not IR	56154	testis expressed 15
ADAM2	8p11.2	CT15	Not IR	2515	ADAM metallopeptidase domain 2
ZNF165	6p21.3	CT53	IR	7718	zinc finger protein 165
CEP55	10q23.33	CT 111	IR	55165	centrosomal protein 55kDa
ATAD2	8q24.13	CT137	IR	29028	ATPase family, AAA domain containing 2
MAGEA3	Xq28	CT1.3	IR	4102	melanoma antigen family A, 3
CTAGE5	14q13.3	CT21.3	IR	4253	CTAGE family, member 5
TTK	6q13-q21	CT96	Co-stim IR	7272	TTK protein kinase
PBK	8p21.2	CT84	Co-stim IR	55872	PDZ binding kinase
PRAME	22q11.22	CT130	Co-stim IR	23532	preferentially expressed antigen in melanoma
CXorf48	Xq26.3	CT55	Co-stim IR	54967	chromosome X open reading frame 48

All CT Antigens used in this study:
ACTL8, ADAM2, ADAM29, AKAP3, AKAP4, ATAD2, BAGE, BRDT, CABYR, CASC5, CCDC33, CCNA1, CEP290, CEP55, CRISP2, CTAG2, CTAGE1, CTAGE5, CTNNA2, CXorf48, DDX43, DKKL1, DMRT1, ELOVL4, GAGE1, GAGE3, GPATCH2, IL13RA2, KIAA0100, LAGE3, LDHC, LUZP4, MAGEA1, MAGEA10, MAGEA11, MAGEA12, MAGEA3, MAGEA4, MAGEA5, MAGEA6, MAGEA8, MAGEB1, MAGEB2, MAGEB3, MAGEB4, MAGEC1, MAGEC2, MAGEC3, MAGED1, MAGED2, MAGEF1, MAGEH1, MORC1, NOL4, NR6A1, NXF2, ODF1, ODF2, OIP5, PAGE1, PAGE4, PBK, PIWIL2, PLAC1, PRAME, PRM1, PRM2, RQCD1, SAGE1, SEMG1, SPA17, SPAG1, SPAG4, SPAG6, SPAG8, SPAG9, SPANXC, SPINLW1, SPO11, SSX1, SSX2, SSX3, SSX5, SYCP1, TAF7L, TDRD1, TEX14, TEX15, TFDP3, THEG, TMEFF1, TPTE, TSGA10, TSP50, TTK, TULP2, VENTXP1, ZNF165.
